# Experimental Studies on the Dynamic Memcapacitance Modulation of the ReO_3_@ReS_2_ Composite Material-Based Diode

**DOI:** 10.3390/nano10112103

**Published:** 2020-10-23

**Authors:** Joanna Borowiec, Mengren Liu, Weizheng Liang, Theo Kreouzis, Adrian J. Bevan, Yi He, Yao Ma, William P. Gillin

**Affiliations:** 1 College of Physics, Sichuan University, Chengdu 610064, China; scu_heyi@126.com (Y.H.); mayao@scu.edu.cn (Y.M.); w.gillin@qmul.ac.uk (W.P.G.); 2 Materials Research Institute and School of Physics and Astronomy, Queen Mary University of London, Mile End Road, London E1 4NS, UK; t.kreouzis@qmul.ac.uk (T.K.); a.j.bevan@qmul.ac.uk (A.J.B.); 3 Sichuan University—Pittsburgh Institute, Chengdu 610207, China; 2017141522059@stu.scu.edu.cn; 4 The Peac Institute of Multiscale Sciences, Chengdu 610031, China; wzliang@pims.ac.cn

**Keywords:** rhenium trioxide, rhenium disulfide, transition metal dichalcogenide, memristor, memcapacitor, nanobattery

## Abstract

In this study, both memcapacitive and memristive characteristics in the composite material based on the rhenium disulfide (ReS_2_) rich in rhenium (VI) oxide (ReO_3_) surface overlayer (ReO_3_@ReS_2_) and in the indium tin oxide (ITO)/ReO_3_@ReS_2_/aluminum (Al) device configuration is presented. Comprehensive experimental analysis of the ReO_3_@ReS_2_ material properties’ dependence on the memcapacitor electrical characteristics was carried out by standard as well as frequency-dependent current–voltage, capacitance–voltage, and conductance–voltage studies. Furthermore, determination of the charge carrier conduction model, charge carrier mobility, density of the trap states, density of the available charge carrier, free-carrier concentration, effective density of states in the conduction band, activation energy of the carrier transport, as well as ion hopping was successfully conducted for the ReO_3_@ReS_2_ based on the experimental data. The ITO/ReO_3_@ReS_2_/Al charge carrier conduction was found to rely on the mixed electronic–ionic processes, involving electrochemical metallization and lattice oxygen atoms migration in response to the externally modulated electric field strength. The chemical potential generated by the electronic–ionic ITO/ReO_3_@ReS_2_/Al resistive memory cell non-equlibrium processes leads to the occurrence of the nanobattery effect. This finding supports the possibility of a nonvolatile memory cell with a new operation principle based on the potential read function.

## 1. Introduction

In 2009, Di Ventra et al. [1] extended the memristor definition, which was first introduced in 1971 by Chua [2], to memcapacitor and meminductor as two newly generalized energy storage components, which are also referred to as memelements. A memcapacitor is a multi-dimensional electronic device with memory and capacitance capability, where its state can be controlled via external stimuli such as electric field (EF) or voltage (*V*). Memcapacitor application as a resistive random access memory (RRAM) is one of the most promising in the field of next-generation nonvolatile memory (NVM), owing to its superior scalability, low power consumption, and high speed. In an ideal memcapacitor, the capacitance depends only on the history of the charge stored on the plates or the voltage across them. The memcapacitor main characteristic is a hysteretic loop, which may or may not pass through the origin [1] when driven by a periodic input. The complex mechanisms of the ionic transport, among other identified mechanisms, inherited in memristive systems introduce new challenges from the perspectives of modeling, characterization, and system architectures. The unique memory characteristics and nanoscale dimensions of the memcapacitor have a wide application prospect in the next-generation nonvolatile memory computing, with multibit high-density data storage without a power source [3]. Moreover, it represents an important research direction aiming to solve the von Neumann bottleneck in modern computers [4]. Memristive devices provide a promising platform to perform biologically inspired computing [5,6], as well as the potential to realize functionalities of neurons and synapses in order to build artificial neural networks [7,8,9]. The memcapacitor application interest, apart from aforementioned, is extended to other areas such as low-pass filters and circuit design [10]. Furthermore, it is suitable for building chaotic circuits for secure communication systems [11,12], which have attracted vast worldwide interest from both academia and industry. In addition, the memcapacitor stimulated considerable interest in the field of nanoionics, since it showed promise for integration with the complementary metal oxide semiconductor (CMOS) technology [13,14]. Apart from information storage and processing, memcapacitors, unlike memristors, can store energy in the form of the EF, therefore opening new possibilities in the concept of the energy storage and redistribution [15,16]. Despite the prospects of innovative application of memcapacitor, it is not yet available commercially in any form. Nonetheless, progress in the field has been mostly achieved in the theoretical work concerning simulation of the memcapacitive circuit models [17,18], with only several reports exhibiting memcapacitive characteristics realized in practical solid-state devices [19,20,21,22,23]. Among them, Chu et al. have reported memcapacitive and memristive properties of a device composed of an Ni-DNA nanowire sandwiched between Au electrodes, where the dual properties were attributed to the change of the Ni oxidation state [19]. Nonvolatile memcapacitance, induced by oxygen ions migration between indium tin oxide (ITO) and the HfO_x_ layer was reported by Park et al. in the developed ITO/HfO_x_/Si metal-oxide semiconductor device [20], and by Yang et al. in a reactive electrode (Mo, Al)/HfO_x_/n-Si structure [21]. In another work of Noh et al. memristive and memcapacitive attributes were demonstrated in the Ti/Pt-Fe_2_O_3_ core–shell nanoparticles/p^+^-Si system as an effect of the nanoparticles potential variation under charging processes [22]. In addition, the memcapacitive property was also realized in a work of Zhao et al. in a new type of photoelectric memcapacitor with a planar Au/La_1.875_Sr_0.125_NiO_4_/Au structure [23]. With reference to the resistance switching phenomenon, it is worth noting that apart from oxygen ions migration, for many systems, it may also involve more complex redox reactions or thermally activated processes [24]. Apart from the aforementioned examples, the limited (at present) number of the practically realized resistance switching memory cells with capacitive properties is caused by the lack of the functional materials, whose capacitance or resistance can be controlled by external variables [25,26,27]. Despite the promising feature of information storage with very low energy loss, the memcapacitive systems still remain the least studied in the class of memelements.

In this study, the material presented is composed of rhenium disulfide (ReS_2_) rich in the surface overlayer formed of rhenium (VI) oxide (ReO_3_) [28]. The ReS_2_ belongs to the class of 2D transition metal dichalcogenides; meanwhile, ReO_3_ is a member of the broad family of transition metal oxides. Recently, they have attracted significant research interest due to their rich physics, which is desirable from the perspective of their successful integration into a number of electronic and optoelectronic devices, such as thin film transistors, solar and fuel cells, photodetectors, and digital logic devices [29,30,31,32,33,34,35]. 

Herein, the memcapacitive ITO/ReO_3_@ReS_2_/Al device characteristics are presented in detail, as follows. The determination of the charge carrier conduction model, quantitative analysis of its electrical properties, as well as an explanation of the resistance switching mechanism, in addition to the frequency modulated capacitance and conductance profile, were successfully carried out based on the experimental data fitting. It was found that resistance switching relies on the electrochemical metallization (ECM) effect and valence change of the sublattice rhenium (Re) atoms, which are induced by migration of the aluminum cations (Al^3+^) and the lattice oxygen (O^2−^) atoms, respectively. Most importantly, the ITO/ReO_3_@ReS_2_/Al electronic–ionic resistive memory cell was controlled by non-equilibrium states (chemical potential generation) evidenced in the occurrence of the nanobattery effect. The establishment of the electromotive force (*emf*) is clearly a function of the chemical and the charge transport processes taking place in the electrochemically active system. 

## 2. Materials and Methods

Fabrication of the ITO/ReO_3_@ReS_2_/Al memcapacitor: First, the substrates of indium tin oxide (ITO, ≈185 ± 5 nm) on a glass (Saide Glass Co. Ltd., Dongguan, China) were patterned using the photolithography method. Prior to the device fabrication, the ITO/glass substrates were cleaned three times in acetone, ethyl, and isopropyl alcohol by treatment in an ultrasonication bath for 10 min and dried in a flow of high-purity N_2_ gas. The water:ethyl alcohol suspension of the ReO_3_@ReS_2_ (*v*/*v* of 1:0.1, concentration 1.73 mg mL^−1^) was prepared by ultrasonication (1 h) and used to form the active layer by the spin-coating process (0.1 mL, 500 rpm, 30 s) on the cleaned ITO/glass substrate. The active material layer thickness was 85.55 ± 3.2 nm. In the final step, the aluminum (Al, Alfa Aesar, Lancashire, UK) top electrode (≈120 ± 4 nm) was deposited using shadow mask by thermal evaporation in a physical vapor deposition (PVD) chamber (Chi-Vac Research and Development Co. Ltd., Dalian, China), under vacuum of 10^−4^ Pa. The acetone, ethyl, and isopropyl alcohol were provided by Keshi Chemical Co. Ltd., Chengdu, China.

Characterization of the ITO/ReO_3_@ReS_2_/Al memcapacitor: Electrical characterization measurements of the fabricated devices were performed with a Keysight B2901A Precision Source Measure Unit (Keysight, Shanghai, China) in air and at ambient temperature (22 ± 1 °C, relative humidity ≈30%), by applying voltage (*V*) and measuring current (*I*) between the top (Al) and the bottom (ITO) electrode. A compliance current (*I*_cc_) of 1 × 10^−2^ A was applied for SET (device switch into ON state) and RESET (device switch into OFF state) processes, respectively, unless otherwise stated. The memcapacitive characteristics of the ITO/ReO_3_@ReS_2_/Al device were examined by recording capacitance–voltage (*C-V*) measurements, by sweeping DC voltage with a superimposed AC signal of 30 mA, on the TTPX Probe Station (Lake Shore Cryotronics, Inc., Westerville, OH, USA) controlled with 4200-SCS Semiconductor Characterization System (Keithley Instruments, Inc., Cleveland, OH, USA). For all the measurements, the voltage was applied to the top electrode, while the bottom electrode was grounded. Impedance spectroscopy measurements were performed on the Metrohm Autolab with PGSTAT30 instrument (Eco Chemie B.V., Utrecht, The Netherlands).

## 3. Results and Discussion

### 3.1. Physicochemical Characterization of the ReO_3_@ReS_2_

The ReO_3_@ReS_2_ solid electrolyte is composed of the rhenium (VI) (Re^6+^ (4f_7/2_, 44.98 eV), oxygen (O^2−^ 1s, 530.59 eV), rhenium (IV) (Re^4+^ 4f_7/2_, 41.85 eV), as well as sulfur (S^2^^−^ 2p_3/2_, 162.96 eV), which is confirmed from the high-resolution X-ray photoelectron spectroscopy (XPS, AXIS UltraDLD, Kratos Analytical Ltd., Manchester, UK) analysis results. Based on the data fitting, the ratio of the rhenium (VI) oxide (ReO_3_) to the rhenium disulfide (ReS_2_) was estimated to be 0.8:1.0 (for details, see Figure S1 in the electronic supplementary material (ESM)). It should be noted that in the ReO_3_ material, the Re atoms are present in the mixed Re^6+^ and Re^7+^ oxidation state; therefore, the ReO_3_ should not be considered as a highly conducting metal oxide, as it is expected from its doped crystal form [36]. Moreover, the transition metal oxides frequently exhibit lattice disorder associated with structural nonstoichiometry [37]. The results from the energy-dispersive X-ray (EDX, FEI Quanta FEG 250, FEI, Hillsboro, OR, USA) spectroscopy confirmed the presence of the Re, S, and O elements in the ReO_3_@ReS_2_ composite material (for details, see Figure S2 in the ESM).

### 3.2. Electronic Characterization of the ITO/ReO_3_@ReS_2_/Al Diode

**Figure 1:**
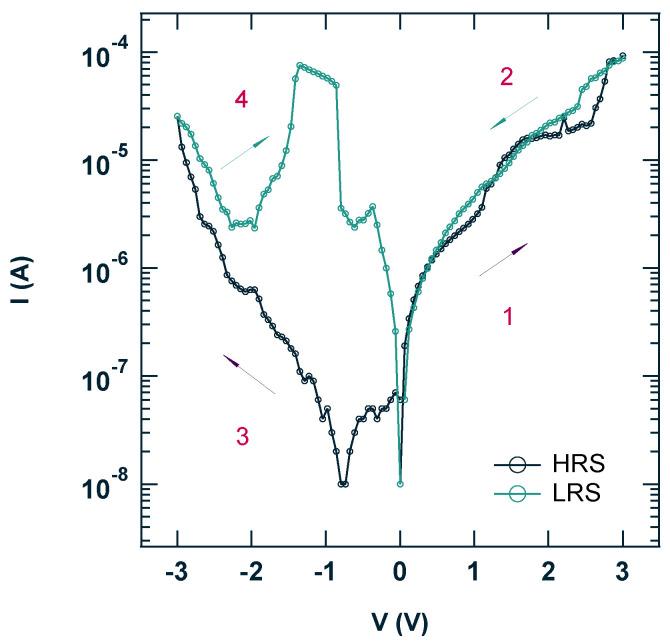
Initial sweep of the ITO/ReO_3_@ReS_2_/Al memcapacitor, measured at a frequency (*f*): 0.6 Hz. Sweeping voltage range: ±3 V, and compliance current (*I*_cc_): 1 × 10^−2^ A. Arrows indicate voltage sweep sequence.

The initial current–voltage (*I-V*) sweep of the ITO/ReO_3_@ReS_2_/Al device presented in Figure 1 exhibits the formation of conductive nanofilaments (CNFs) taking place at low bias voltage, *V*_SET, *form*_ of 2.57 ± 0.06 V. The abrupt bipolar resistance switching between the high (HRS) and low (LRS) resistance state, in the forward (FB) as well as in the reverse bias (RB) sweep direction, is typically observed in the case of induced memristance or redox reaction driven devices [38]. The significant variation in the resistance and the operating voltages with a consecutive voltage sweep can be ascribed to the random redistribution and incorporation of the mobile ions in the ReO_3_@ReS_2_ composite material network under an external electric field (EF) (see Figure 1).

**Figure 2:**
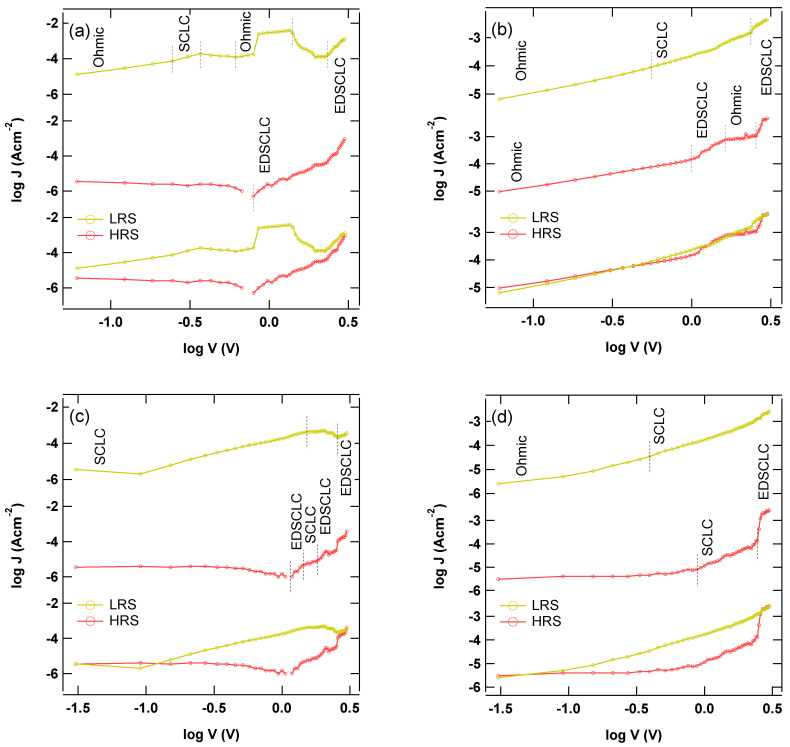
Plot of log *J* vs. log *V* of the ITO/ReO_3_@ReS_2_/Al for the initial scan under (**a**) negative and (**b**) positive bias; and for the fifth consecutive scan under (**c**) negative and (**d**) positive bias. Sweeping voltage range: ±3 V, *I*_cc_: 1 × 10 ^−2^ A, and *f*: 0.6 Hz.

The space charge limited current (SCLC) conduction model of the charge carrier, shown in Figure 2a,b (initial scan) as identified from the appearance of the ohmic regions (*I*α*V*, FB sweep), points on the formation of the CNFs within the ReO_3_@ReS_2_ electrolyte layer [39].

The ohmic conduction (LRS under FB, *V* < *V*_TFL_ = 0.55 V, ITO bottom electrode (BE) injection) is accompanied by the filling processes of the trap states by the injected charge carrier at the ITO/ReO_3_@ReS_2_ interface, which is evidenced in the occurrence of the SCLC. The third region involves filled trap states, which are exponentially distributed (ED) in energy within the forbidden band gap (*V*_TR_ = 2.39 V and *I*α*V*^4^). The Gaussian distribution function of the trap states (see Equation (S1) in the ESM) can be associated with the statistical dispersion of the charge carrier polarization energy, due to the structural irregularities of the material lattice [40]. The observation of the second ohmic region at higher voltages (*V* > 0.62 V in the HRS/LRS under FB and RB sweep) is attributed to the dominated bulk ReO_3_@ReS_2_ conductivity. The hysteresis curve of the fifth consecutive *I–V* sweep is equally represented by the SCLC conduction model (see Figure 2c,d). Under consecutive voltage sweeps, from the initial to the fifth scan, the memcapacitor electrical properties underwent observable change. The dynamic processes taking place in the device resulted in the decrease of the charge carrier mobility (*μ*, from 6.95 × 10^−11^ to 5.11 × 10^−11^ cm^2^V^−1^s^−1^) carrier concentration in thermal equilibrium (*n*_o,_ from 1.24 × 10^20^ to 4.28 × 10^19^ cm^−3^), number of trap states (*N*_t_, from 7.93 × 10^20^ to 3.79 × 10^20^ cm^−3^), and free-carrier concentration (*n*, from 9.11 × 10^19^ to 4.08 × 10^19^ cm^−3^), as well as the effective density of states in the conduction band (*N*_c_, from 4.84 × 10^14^ to 1.78 × 10^14^ cm^−3^). Corresponding results from the evaluation of the electric properties of the device attained from the experimental data fitting are listed in Table S1 in the ESM.

**Scheme 1:**
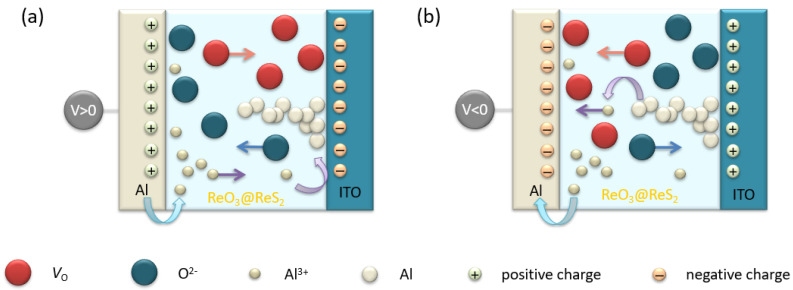
Schematic representation of the ion redistribution within the ITO/ReO_3_@ReS_2_/Al under (**a**) forward (V > 0) and (**b**) reverse (V < 0) bias voltage.

The low carrier mobility (*µ*) of the order of 10^−11^ cm^2^V^−1^s^−1^ is an indication of the mixed electronic–ionic conduction of the charge carrier in the ReO_3_@ReS_2_ electrolyte (for details of the charge carrier conduction, please see Figure 2 and Table S1). The bipolar switching mode is attributed to the electrochemical redox reactions and the high solubility of the Al^3+^ and O^2−^ ions in the ReO_3_@ReS_2_ material network. The inversely proportional surface area dependence on the memcapacitor resistance (*R*, see Figure S3 in the ESM) implies that the CNF-type resistance switching is not the dominant effect in the ITO/ReO_3_@ReS_2_/Al, but it rather occurs over the whole area of the cell interface [27,41]. Under FB (positively biased top electrode (TE)), the EF-induced O^2-^ migration (drift and diffusion) in the lattice sites leads to the consecutive growth of the oxygen vacancy (*V*_o_) channels expanding toward the anode. Simultaneously, the oxidation process of the transition metal cations (Re^6+^) in the Al electrode vicinity prevents the *V*_O_ channels from short-circuiting the electrodes [42]. In the HRS, the oxygen-deficient channels serve as the active sites for the Al^3+^ ions precipitation and subsequent growth of the highly conductive metal NFs, propagating in the direction from the anodic to the cathodic interface (see Equation (S2) in the ESM and Scheme 1a) [43]. The ITO/ReO_3_@ReS_2_/Al resistance switching is driven by the ECM process [24]. The mobile Al^3+^, drifting in the ion conducting ReO_3_@ReS_2_ layer, is formed under anodic dissolution of the active Al TE, which is experimentally verified by the temperature-depressed conductivity of the ITO/ReO_3_@ReS_2_/Al (as evidenced by the derived Al resistance temperature coefficient (*α*) of 3.9 × 10^−3^ K^−1^ being in agreement with the theoretical value, see Figure S4 in the ESM) [44]. The less mobile Re^6+^ cations compensate the oxygen deficiency by trapping electrons released from the cathode (see Equation (S3) in the ESM), where the electrons fill up the empty states of the Re 5*d* band. Furthermore, the drift of the O^2-^ toward the cathode gives rise to the evolution of molecular oxygen (O_2(g)_, see Equation (S4) in the ESM), which is revealed through the formation of surface bubbles (see Figure S5 in the ESM). The O^2−^ depletion (ReO_3-*x*_, where *x* is the number of the O^2−^) generates a high number of *V*_O_, which are considered to act as the effective donors in the n-type oxide semiconductors [45]. This process is portrayed through an initial decrease of the electrolyte/BE contact resistance (gradual increase of the HRS current, see Figure S6 in the ESM) with consecutive voltage sweeps (maximum EF of 5.8 × 10^7^ Vm^−1^), due to the depletion of the available interfacial O^2−^ at the electron injecting electrode interface. Finally, the reset of the cell into the OFF state is completed under RB polarity sweep, which induces the electrochemical dissolution of the top parts of the Al CNFs (see Scheme 1b).

**Figure 3:**
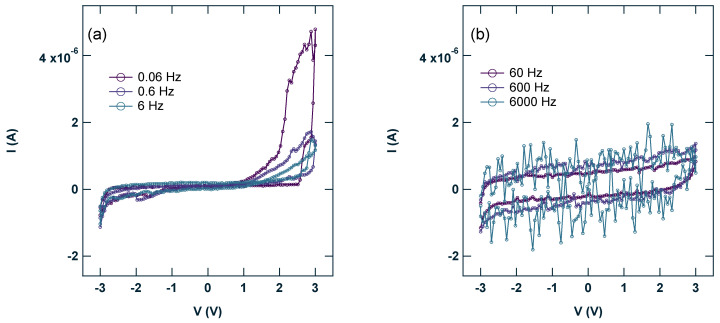
Plot of *I* vs. *V* as a function of scan frequency in the range of (**a**) 0.06–6 Hz and (**b**) 60–6000 Hz. Sweeping voltage range: ±3 V, and *I*_cc_: 1 × 10^−2^ A.

**Figure 4:**
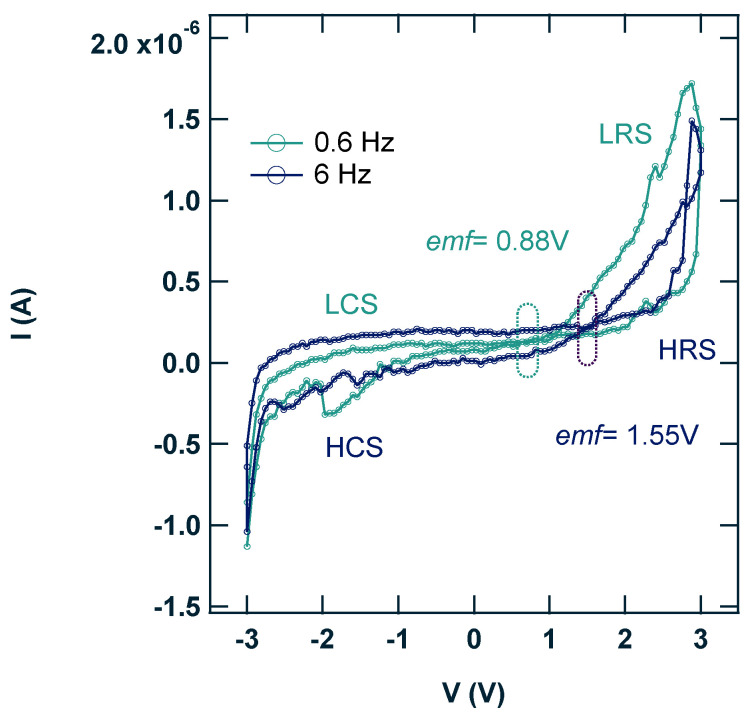
The *I* vs. *V* dependence on the scan frequency at 0.6 and 6 Hz of the ITO/ReO_3_@ReS_2_/Al. The sweeping voltage range: ±3 V, and *I*_cc_: 1 × 10^−2^ A.

The experimentally validated EF-driven ion transport was found to be in agreement with the thermally induced ion hopping in the FB regime (Mott and Gurney model, see Equation (S5) in the ESM) [46]. The activation energy ($W_{a}^{0}$, at 295 K) for the hopping in the absence of EF was estimated to be 0.34 (y_(1)_) and 0.27 eV (y_(2)_) in HRS and LRS, respectively (ln *J*α*E* regime, see Figure S7 in the ESM), which is comparable to the average $W_{a}^{0}$ for the electron hopping. Furthermore, the electrons emitted from the trap states in the thermally activated Poole–Frenkel (P-F) process were also found to contribute to the charge carrier conduction (ln *JE^−1^* vs. *E^1/2^* plot, see Figure S8 in the ESM). The barrier height of the traps (*q*φ*_T_*), derived from the fitting of the P-F plot was equal to 0.69 ± 0.01 eV. The temperature-dependent activation energy (*E*_a_) for the carrier transport of the ITO/ReO_3_@ReS_2_/Al (ln *J* vs. *k_b_*^−1^*T*^−1^, see Figure S9 in the ESM) exhibited two regions: the charge-hopping conduction in the ReO_3_@ReS_2_ framework at lower temperatures (*T* < 34.2 K), comprising the diffusion of carrier between existing vacancy and/or interstitial sites (*E*_a_ = −0.51 eV, extrinsic region), and, as expected for ionic conductors, carrier diffusion and simultaneous processess of formation of new vacancy sites (*E*_a_ = 0.13 eV, intrinsic region) taking place at elevated temperatures (*T* > 34.2 K). The dynamic control over the mobile ions redistribution within the electrolyte network, achieved through adequate selection of the EF strength as well as the number of the voltage sweep cycles (see Figure 1, and Figure S6 in the ESM), can effectively impact the memcapacitor electronic state [27]. The memristor resistance, after five consecutive voltage sweeps (Δ*V:* ±3 V), was demonstrated to be in agreement with the characteristic shrinking of the device hysteresis loop with rise of the scan frequency (*f*), as a consequence of a slow response of the charge carrier to the high-frequency EF oscillation (FB, see Figure 3a) [2]. However, the principle of the zero-crossing hysteresis of the ITO/ReO_3_@ReS_2_/Al is violated due to the establishment of the chemical potential gradient (nanobattery) within the device active layer [47]. At frequencies of 0.6 and 6Hz, the generated electromotive force (*emf*, see Figure 4) resulted in the cell voltage (*V*_emf_) reaching values of 0.88 and 1.55 V, respectively.

The appearance of the *emf*, which has been reported in several devices [48,49], justifies the necessity of the expansion of the memristive theory to account for the extended memcapacitive and meminductive elements observed in practical memristive devices [1,48,50]. The presence of the active element is clearly a function of the device non-equilibrium state, which is illustrated by inhomogeneous charge redistribution within the active layer, and its dynamic response to the varied frequency modulation. These processes generating the *emf* in the ITO/ReO_3_@ReS_2_/Al memcapacitor can be associated either with the concentration gradient between Al^3+^ and the majority charge carrier and/or the Al^3+^ potential difference between the TE and CNF [50]. However, at frequencies above 6 Hz, the current trend follows the open circuit voltage (OCV) hysteresis, which is typical for capacitors (see Figure 3b).

Moreover, the dynamic extraction of the O^2-^ from the ReO_3_@ReS_2_ toward the Al electrode under FB sweep [51,52] decreased the electrolyte relative permittivity (*ε*_r, initial_ ≈ 220 to *ε*_r, final_ ≈ 36) and in effect caused a variance in the device capacitance (*C*, decrease of 83.5% where *C*_initial_ = 45.5 nF), which is restrained by the active material dielectric constant. The capacitance modulation is strictly a function of the mean Schottky barrier width (*W*_d_) as well as the chemical potential gradient; however, it is independent from the formation and rupture of the CNFs [53]. The applied BV level not only modulates the structural properties of the material but also the corresponding active layer/electrode interfaces, which is portrayed through the dynamic variation of the current with consecutive number of sweeping cycles (see Figures S6 and S10 in the ESM). The Schottky barrier (*q*φ*_b_*) profile of the electrolyte/electrode junction can be electrically modulated either by movement of the vacancies away/toward the cell interface by applying reversely polarized bias stress [54], or by carrier trapping/detrapping at defect sites near the junction interface [23]. The process of filling off the interface trap states with injected electrons (HR and LR state in FB, see Figure S10 in the ESM) increases the Fermi level (*E_F_*) and effectively diminishes the *q*φ*_b_* for electron injection. The *C-V* curves at the electrode interfaces show hysteretic behavior (see Figure S11 in the ESM), where the *W*_d_ is narrower in the LRS, facilitating the electrons tunneling through the thin Schottky barrier (this is in agreement with the positive shift of the flat-band voltage, Δ*V*_FB_, as discussed below) [55]. This mechanism has been proven to be also valid for devices based on perovskite materials [56]. Significantly increased ITO/ReO_3_@ReS_2_/Al rectification (FB range) was observed under enhanced *EF* strength up to 8.2 × 10^7^ Vm^−1^ (ΔV: ±7 V, see Figure S12 in the ESM). The elevated EF induced motion of the O^2-^ (better described by the migration of *V*_O_), which in turn triggers the electrolyte reconfiguration and extension of the conducting channels, finally yields device electrode bridging (*R* = 6.35 × 10^−3^ Ω in FB, see Figure S12 in the ESM).

**Figure 5:**
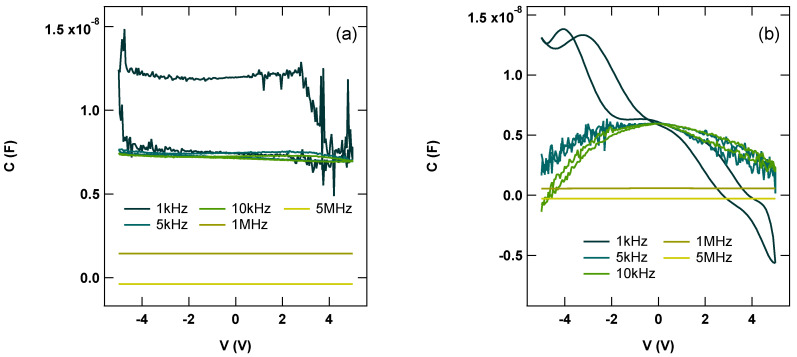
The *C* vs. *V* curves of the ITO/ReO_3_@ReS_2_/Al in the frequency range of 1 kHz–5 MHz for (**a**) standard and (**b**) short-circuited devices. In (**b**), the *C-V* curve at a scan frequency of 1 kHz was fitted with a polynomial function (original data can be found in Figure S13). Higher resolution *C*-*V* curves acquired at frequencies from 1 kHz to 5 MHz (**a**), and at 1 and 5 MHz (**b**) are shown in Figure S14. Sweeping voltage range: ±5 V, and *I*_cc_: 1 × 10^−2^ A.

With reference to the aforementioned mechanisms, the ITO/ReO_3_@ReS_2_/Al memristor *C-V* dependence on the scan frequency (*f*_AC_) relies strongly on the device interface profile and bulk distribution of the charge carrier and trap states (see Figure 5) [57]. Under exertion of the large negative BV (−20V < *V* < *V*_T_ and *f* = 1kHz), the accumulation of the large number of the minority carriers (*V*_O_) at the electrolyte/TE interface, exceeding the number of the dopant carrier, gives rise to strong surface inversion (*C-V* plot, see Figure 5a and Scheme S1 in the ESM).

A large capacitance is formed due to the accumulation of the incremental charge at the inversion layer of the semiconductor (*C* = 1.20 ± 0.02 × 10^−8^ F), which remains in the equilibrium state irrespective of the level of the applied voltage. A subsequent increase of the potential (*V* > *V*_T_) induces the depletion of the minority carrier from the inversion region, leading to the generation of the depletion layer (*E*_i_ crosses below *E*_F_, see Scheme S1 and Equation (S6) in the ESM). As expected, the highest capacitance dependence on the scan frequency occurs in the strong inversion regime. The inversion layer is not formed at high frequencies (*f* ≥ 5 kHz, non-equilibrium state), where the minority carriers are not able to respond to the applied external high-frequency AC signal, and the growing charge number is kept at the edge of the depletion region. Under these circumstances, the depletion layer width (*W*_dl_) keeps increasing beyond its maximum thermal equilibrium value, leading to a capacitance that further decreases with voltage (see Equation (S7) in the ESM). Importantly, the strong positive shift of the flat-band voltage (Δ*V*_FB_ ~2.85 V) observed for the *C-V* sweep acquired at frequency of 1 kHz (see Figure 5a) is a clear indication of the process of trapping of the electrons injected from the electrode [58].

**Figure 6:**
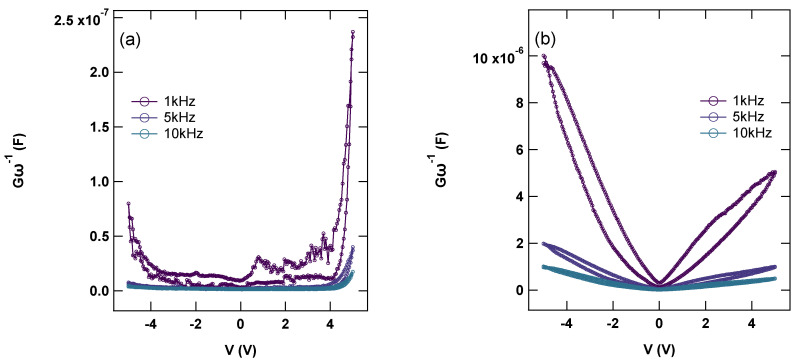
The *Gω*^−1^ vs. *V* sweep of the ITO/ReO_3_@ReS_2_/Al memcapacitor for (**a**) standard and (**b**) short-circuited device. Voltage sweep direction from −V to +V, *I*_cc_: 1 × 10^−2^ A.

Interestingly, the appearance of the negative capacitance, in both the FB and RB region, was observed after short-circuiting the device electrodes (see Figure 5b). The increase of the frequency as well as the BV lead to a capacitance drop in the FB region. The rise of the voltage generated an instant injection of both majority and minority charge carriers into the device active layer (influenced by the properties of the injecting contact and the solid electrolyte charging processes). The following slow redistribution of the minority carrier (slower *V*_O_ diffusion in comparison to the drift of the electrons) within the ReO_3_@ReS_2_ brought on the decrease of the current and, in effect, influenced the change of the diffusion capacitance of the system, as well as caused the appearance of the inductive effect at low frequencies [59]. This mechanism was identified to be in agreement with the drop of the ITO/ReO_3_@ReS_2_/Al conductance (normalized with angular frequency, *Gω*^−1^) with a subsequent rise of the BV (see Figure 6).

The aforementioned mechanism is supported by the low series resistance of the device (see Figure S15 in the ESM). Moreover, at low voltages, the *G* of the memristor is low, and the transient current is dominated by the displacement component related to the release and escape of the electrons from the traps, recharging processes, and injection properties of the emitter barrier [57]. The decrease of the inversion layer capacitance (RB region) can be understood from the perspective of widening of the *W*_dl_ with rise of the scan frequency, which is caused by the minority charge depletion. In addition, the constant value of the capacitance at 0 V bias (*C* = 6.02 ± 0.05 × 10^−6^ F), irrespective of the frequency, is more likely to be associated with the device geometric capacitance than the charging of the interface states [60]. Nonetheless, the heating effects causing a drop of the device resistance or charge carrier generation from the CNF dissolution might be considered as other probable explanations of the phenomenon.

## 4. Conclusions

In conclusion, reliable and controllable memcapacitive behavior was investigated in the two-terminal device structure utilizing ReO_3_@ReS_2_ as a solid electrolyte. The electronic–ionic conduction, with the charge carrier mobility of 10^−11^ cm^2^V^−1^s^−1^, was identified in the memcapacitor from the SCLC model fitting. It was shown that a moderate rise of the BV across the active layer can generate a large enough EF to achieve the control over the device electronic state. The origin of the occurrence of the *emf*, as high as 1.55 V, was associated with the frequency-modulated device charge carrier dynamic state variation. Furthermore, the experimental work presented here indicates a strong contribution to the concept of practical memcapacitor modeling and most importantly device structure engineering, from the perspective of understanding the composition of the ionic materials utilized for practical devices fabrication. The chemical potential generated by the electronic–ionic ITO/ReO_3_@ReS_2_/Al memcapacitor non-equlibrium processes opens up new possibilities toward memory cells whose operation relies on the potential read function.

## Supplementary Materials

The following are available online at https://www.mdpi.com/2079-4991/10/11/2103/s1, Figure S1: High resolution XPS spectra, Figure S2: SEM and EDX spectra, Table S1: Electronic properties of the ITO/ReO_3_@ReS_2_/Al, Figure S3: Device area dependent *I* vs. *V* plot, Figure S4: Normalized resistance (*RR*_o_^−1^) vs. temperature (*T-T*^o^) plot, Figure S5: SEM image of the ITO/ReO_3_@ReS_2_/Al device, Figure S6: The *I* vs. *V* plot in the voltage range ±5 V, Figure S7: The ln *J* vs. *E* plot, Figure S8: The ln *JE*^−1^ vs. *E*^1/2^ plot, Figure S9: The ln *J* vs. *k_b_*^−1^*T*^−1^ Arrhenius plot, Figure S10: The ln *IT*^−2^ vs. *V*^1/2^ plot, Figure S11: The *C* vs. *V* plot, Figure S12: The *I* vs. *V* plot in the voltage range ±7 V, Scheme S1: Energy band and charge density diagram, Figure S13: The *C* vs. *V* plot at scan frequency of 1 kHz, Figure S14: The *C* vs. *V* plot, Figure S15: Nyquist plot of the impedance spectra.
