# Supplementary material for: Experimental Studies on the Dynamic Memcapacitance Modulation of the ReO3@ReS2 Composite Material-Based Diode
Source: Nanomaterials (Basel). 2020 Oct 23;10(11):2103. doi: 10.3390/nano10112103 (PMC7690752; doi:10.3390/nano10112103)
Supplement: Supplementary file 1 [file nanomaterials-10-02103-s001.pdf]

nanomaterials

Article

Experimental Studies on the Dynamic Memcapacitance Modulation of the $ReO_3@ReS_2$ Composite Material-Based Diode

Joanna Borowiec [1,2,*], Mengren Liu [3], Weizheng Liang [4], Theo Kreouzis [2], Adrian J. Bevan [2], Yi He [1], Yao Ma [1] and William P. Gillin [1,2]

1 College of Physics, Sichuan University, Chengdu 610064, China; scu_heyi@126.com (Y.H.); mayao@scu.edu.cn (Y.M.); w.gillin@qmul.ac.uk (W.P.G.)
2 Materials Research Institute and School of Physics and Astronomy, Queen Mary University of London, Mile End Road, London E1 4NS, UK; t.kreouzis@qmul.ac.uk (T.K.); a.j.bevan@qmul.ac.uk (A.J.B.)
3 Sichuan University—Pittsburgh Institute, Chengdu 610207, China; 2017141522059@stu.scu.edu.cn
4 The Peac Institute of Multiscale Sciences, Chengdu 610031, China; wzliang@pims.ac.cn
* Correspondence: borowiec@scu.edu.cn; Tel.: +86-028-854-12323

1. Materials and Methods

The scanning electron microscopy (SEM) measurements were taken with a JSM-7500F and FEI Quanta 250 FEG scanning electron microscopes operated at 10–20 keV. X-ray photoelectron spectroscopy (XPS) measurements were conducted using an AXIS Ultra^{DLD} instrument (Kratos, UK). All data were acquired using monochromatic Al K_α X-rays (1486.6 eV), with a pass energy of 25 eV. No considerable sample charging was observed. Charge referencing was done against adventitious carbon (C 1s, 284.6 eV). Spectra are presented with intensity in counts per second (CPS) without smoothing, for which a Tougaard-type background was subtracted. Fitting of the experimental peaks were obtained using combinations of Gaussian/Lorentzian lines, with the 70/30 proportion. Spin-orbit splitting ratios of 2:1 for the sulfur $2p_{3/2}$ to $2p_{1/2}$, and 4:3 for rhenium $4f_{7/2}$ to $4f_{5/2}$ doublets, with the corresponding separation in binding energy of 1.16 and 2.40 eV were implemented. Relative atomic concentrations were calculated considering peak areas and atomic sensitivity factors (ASFs) provided by instrument manufacturer.

2. Experimental results

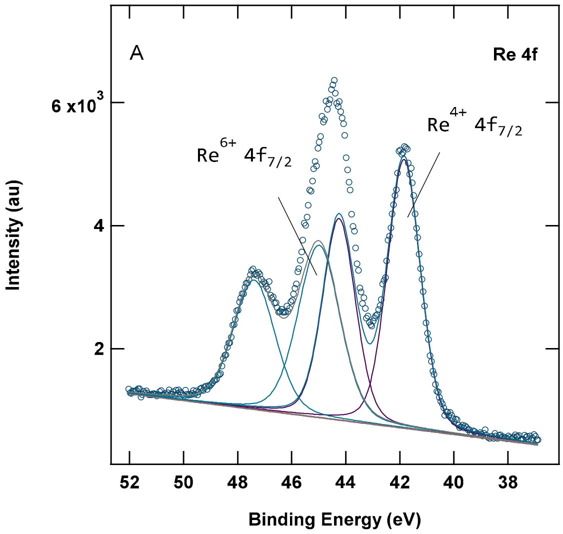

(a)

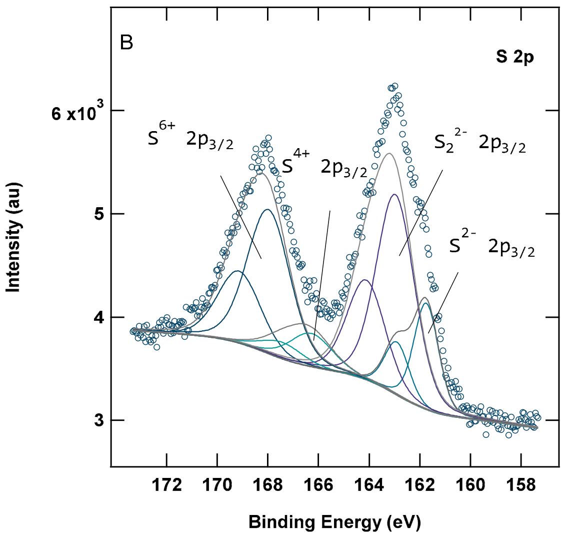

(b)

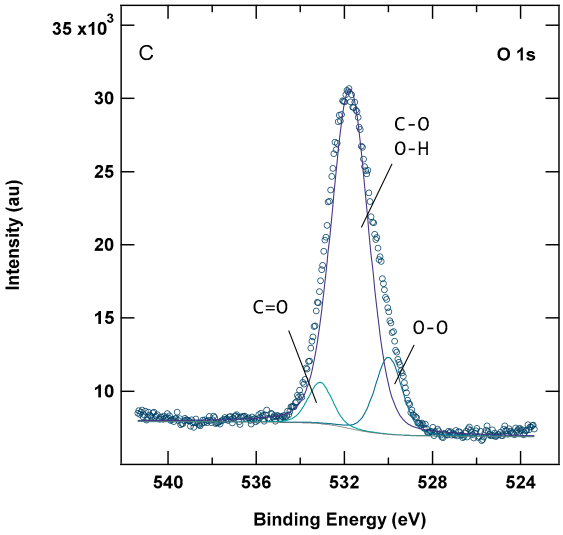

(c)

Figure S1. High resolution XPS spectra of (**a**) Re 4f, (**b**) S 2p, and (**c**) O 1s recorded at emission angle normal to the sample surface.

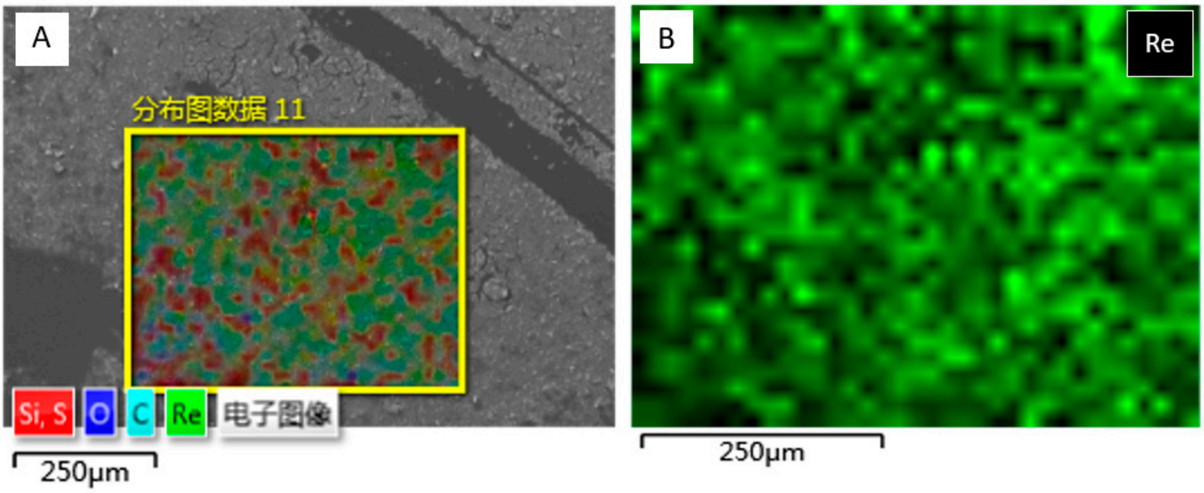

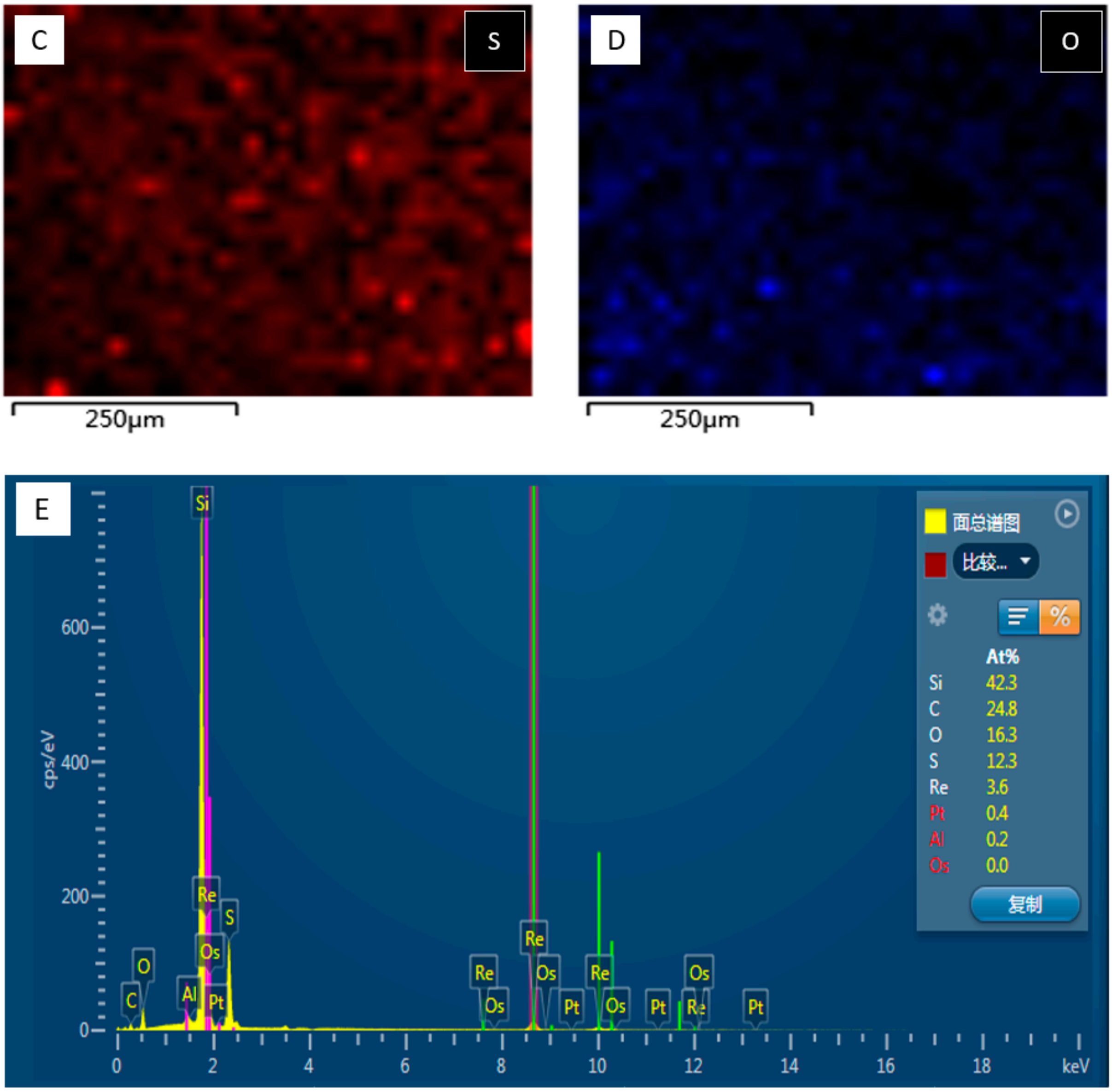

Figure S2. SEM image of the $ReO_3@ReS_2$ indicating the area (yellow rectangle) of EDX data acquisition (**a**) with elemental mapping of Re (**b**), S (**c**), and O (**d**). EDX spectrum obtained from the area shown in (**a**) with atomic percentages of the elements.

2.1. Equation S1

Gaussian function of the ED SCLC [6–8]:

$$J = \mu_0 N_c q^{1-n} \left(\frac{\varepsilon_0 \varepsilon_r n}{N_t(n+1)}\right)^n \left(\frac{2n+1}{n+1}\right)^{n+1} \frac{V^{n+1}}{d^{2n+1}} \quad (1)$$

where,

J	current density
μ_0	free carrier mobility
N_c	effective density of states in the conduction band
q	electron charge

$\varepsilon 0 \varepsilon_r$		dielectric constant
N_t		trap concentration
n	$\frac{T_c}{T}$, where T_c is distribution parameter, and T is absolute temperature	
d	film thickness	

Table S1. Electronic properties of the ITO/ReO_3@ReS_2/Al after initial and 5th consecutive voltage sweep.

Parameter	Initial Voltage Sweep	5th Consecutive Voltage Sweep
Charge carrier mobility (μ)	6.95×10^{-11} cm^2 V^{-1} s^{-1}	5.11×10^{-11} cm^2 V^{-11} s^{-1}
Carrier concentration in thermal equilibrium (n_o)	1.24×10^{20} cm^{-3}	4.28×10^{19} cm^{-3}
Trap concentration (N_t)	7.93×10^{20} cm^{-3}	3.79×10^{20} cm^{-3}
Free-carrier concentration (n)	9.11×10^{19} cm^{-3}	4.08×10^{19} cm^{-3}
Ratio of carrier density to total carrier (θ)	1.21	0.93
Effective density of states in the conduction band (N_c)	4.84×10^{14} cm^{-3}	1.78×10^{14} cm^{-3}

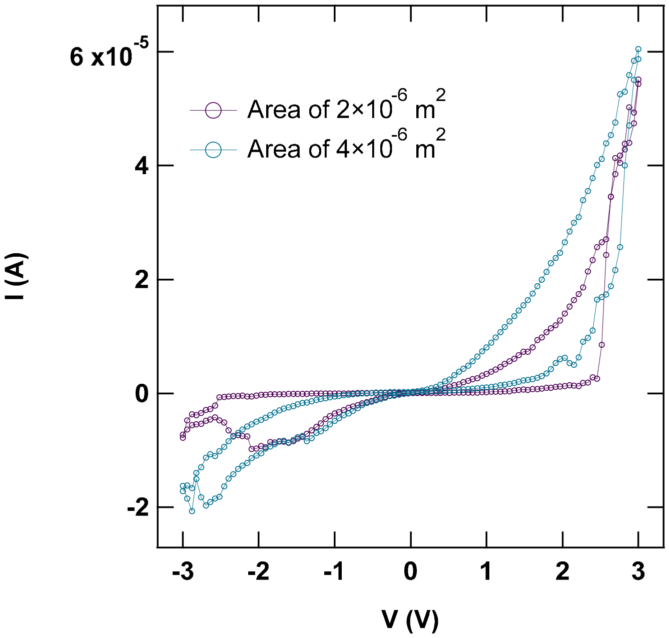

Figure S3. Device active area dependent *I* vs *V* characteristics of the ITO/ReO_3@ReS_2/Al device for the third consecutive voltage sweep. Sweeping voltage range: ±3 V, I_{cc}: 1×10^{-2} A, and *f*: 0.6 Hz.

2.2. Equation S2

Aluminum reduction reaction:

$$Al^{3+} + 3e^- \rightarrow Al^0 \quad (2)$$

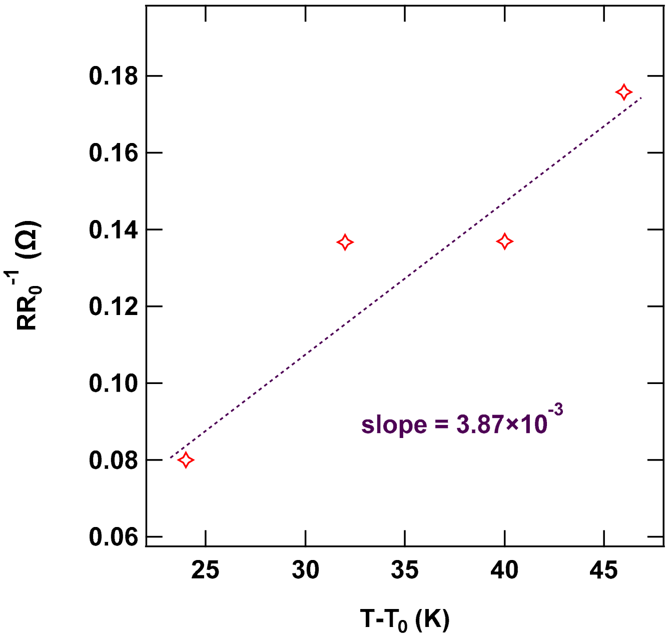

Figure S4. Normalized resistance (RR_o^{-1}) vs temperature (T-T_o) of the the ITO/ReO_3@ReS_2/Al memcapacitor. Dashed lines represent linear data fit.

2.3. Equation S3

Rhenium reduction reaction:

$$ne + Re^{6+} \longrightarrow Re^{(6-n)+} \tag{3}$$

where,

n number of electrons

2.4. Equation S4

Oxygen evolution reaction:

$$O_o \longrightarrow V_o + \frac{1}{2}O_{(g)} + 2e^- \tag{4}$$

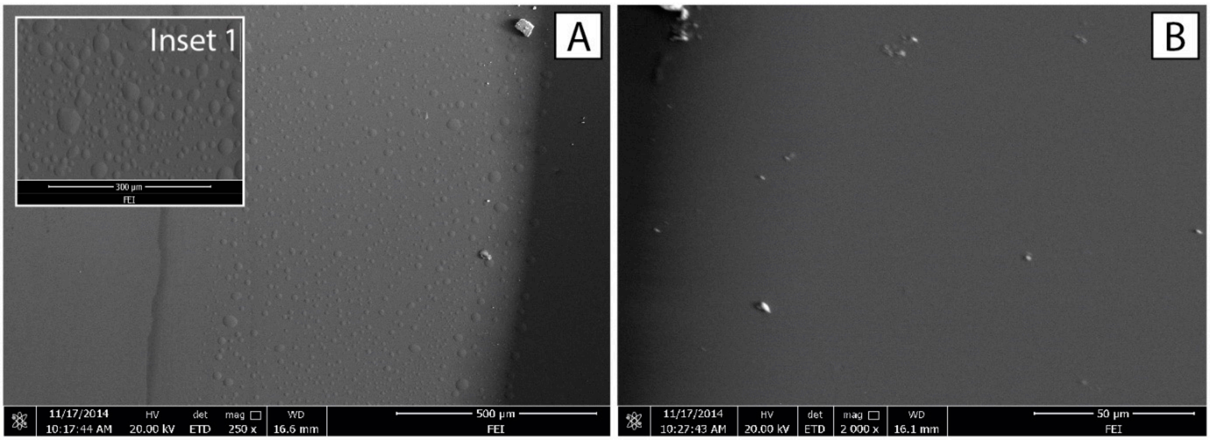

Figure S5. SEM images of the ITO/ReO_3@ReS_2/Al memcapacitor after (**A**) consecutive voltage sweeps and (**B**) pristine device. Inset 1: High magnification SEM image of (**A**).

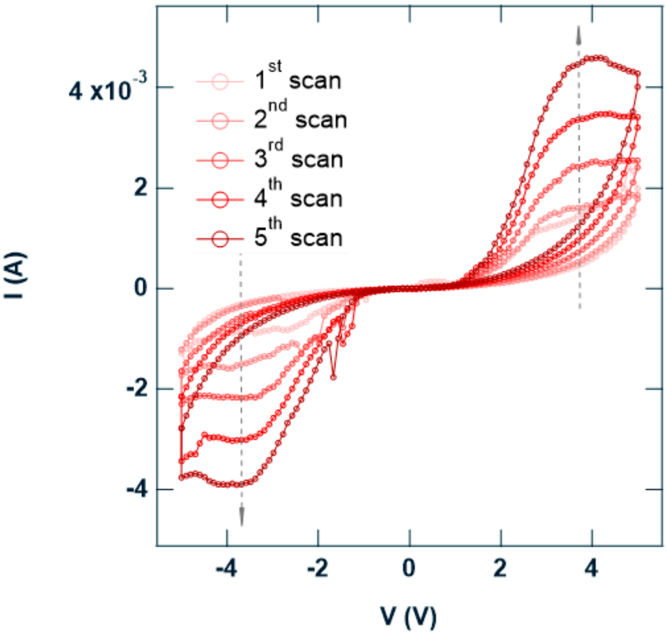

Figure S6. The *I* vs *V* sweep of the ITO/ReO_3@ReS_2/Al in the rage of ±5 V. Voltage sweep direction from -V to +V, I_{cc}: $1{\times}10^{-2}$ A, and *f*: 0.6 Hz.

2.5. Equation S5

Mott-Gurney equation for low electric fields ($E \ll kT/aze$):

$$J = \frac{(ze)^2 cE}{kT} a^2 \nu \, exp \left(\frac{W_a^0}{kT}\right) = \frac{(ze)^2 cD\Delta\varphi_{SE}}{kTd} \quad (5)$$

where,

J ionic current density

c concentration of mobile ions

a hopping distance

ν hopping frequency

D diffusion coefficient

$\Delta_{\varphi_{SE}}$ potential drop across the ion-conducting film

E electric field strength

W_a^0 energy barrier

d ion-conducting film thickness

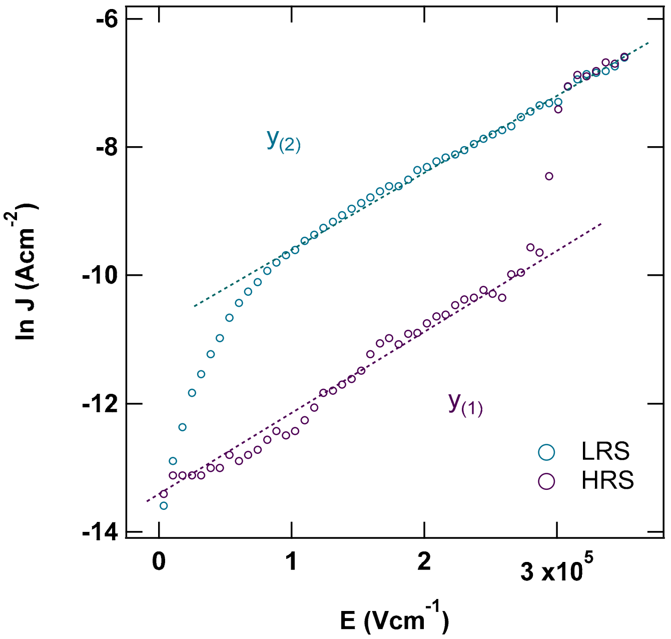

Figure S7. Plot of ln *J* vs *E* of the ITO/ReO3@ReS2/Al for the 5th voltage sweep under forward bias. Sweeping voltage range from 0 to 3 V, I_{cc}: 1×10−2 A, and *f*: 0.6 Hz. Dashed lines represent linear data fit.

The ion hopping conduction model experimental data fitting gave the linear dependence of ln *J* vs. *E* of: $y_{(1)} = 1.36\times10^{-5}$ ($\pm2.57\times10^{-7}$)x – 13.56 (±0.04), and $y_{(2)} = 9.93\times10^{-6}$ ($\pm1.26\times10^{-6}$)x – 10.07 (±0.41), as presented in Figure S8.

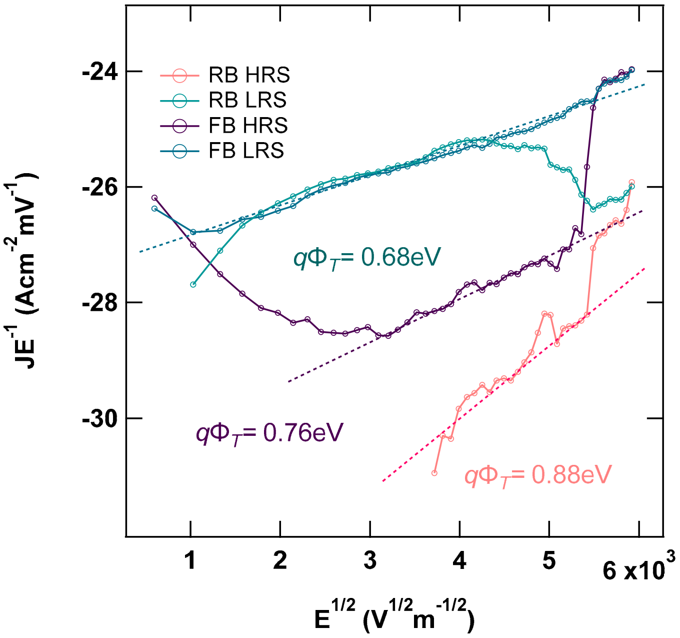

Figure S8. Plot of the ln JE^{-1} vs $E^{1/2}$ of the ITO/ReO3@ReS2/Al memcapacitor. Sweeping voltage range: ±3 V, I_{cc}: 1×10−2 A, and *f*: 0.6 Hz.

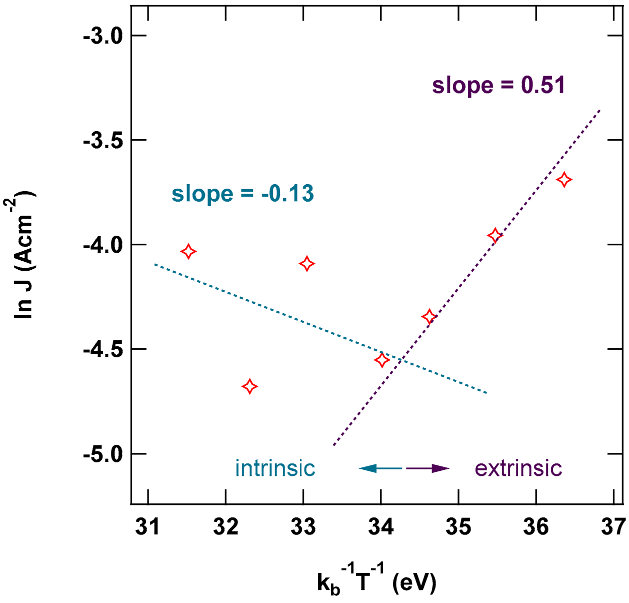

Figure S9. Arrhenius plot of ln *J* vs $k_b^{-1}T^{-1}$ of the temperature dependent current of the ITO/ReO_3@ReS_2/Al memcapacitor. Sweeping voltage range: ±5 V, I_{cc}: 1 × 10^{-2} A, and *f*: 0.6 Hz.

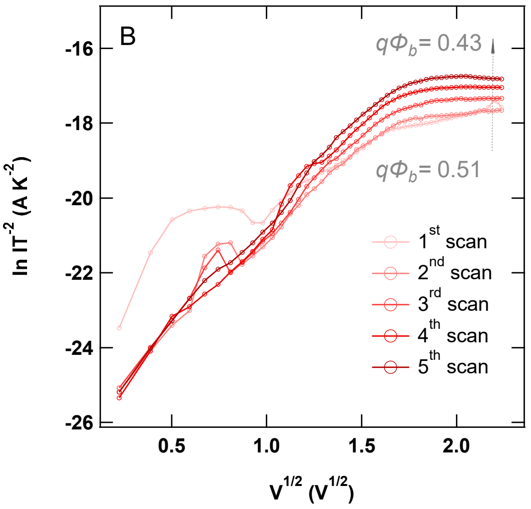

(a)

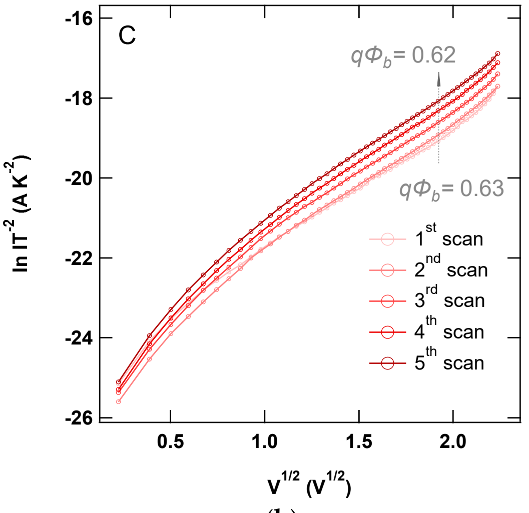

(b)

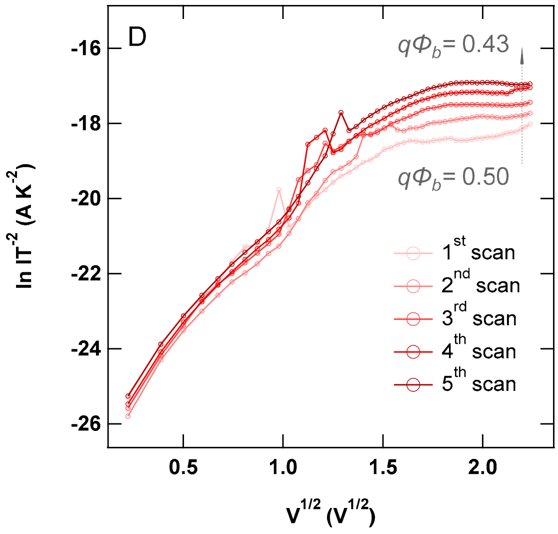

(c)

Figure S10. Plot of ln (I/T^2) vs $V^{1/2}$ of ITO/ReO_3@ReS_2/Al for (a) negative HRS, (b) positive HRS, (c) positive LRS and (d) negative LRS. Sweeping voltage range: ±5 V, I_{cc}: 1×10⁻² A, and f: 0.6 Hz.

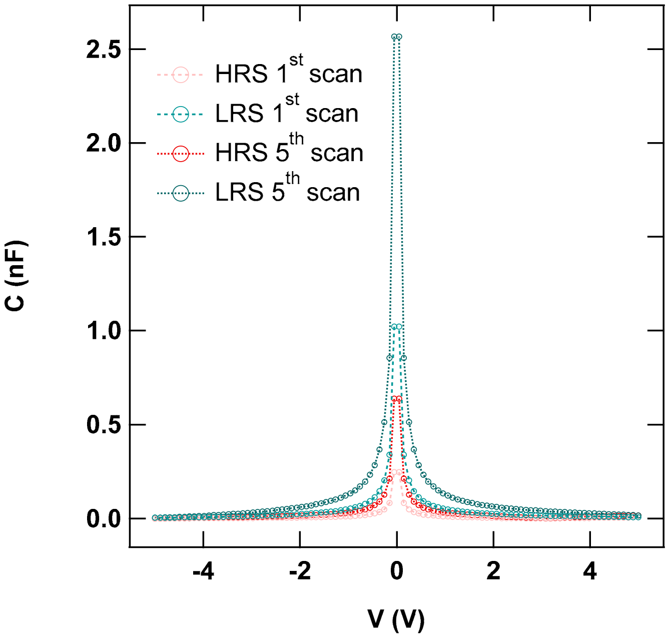

Figure S11. The C vs V curves of the ITO/ReO_3@ReS_2/Al memcapacitor. Sweeping voltage range: ±5 V, I_{cc}: 1×10^{-2} A, and f: 0.6 Hz.

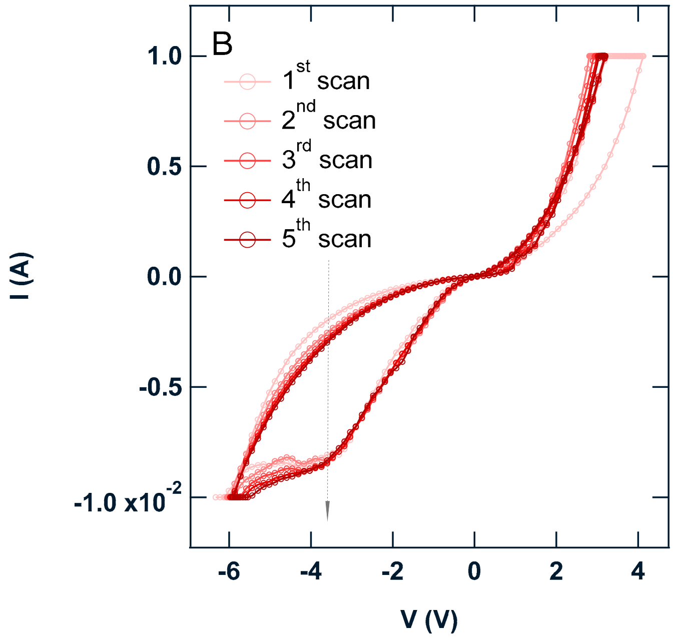

Figure S12. The *I* vs *V* sweep of the ITO/ReO_3@ReS_2/Al in the rage of ±7 V. Voltage sweep direction from -V to +V, I_{cc}: 1×10^{-2} A, and *f*: 0.6 Hz.

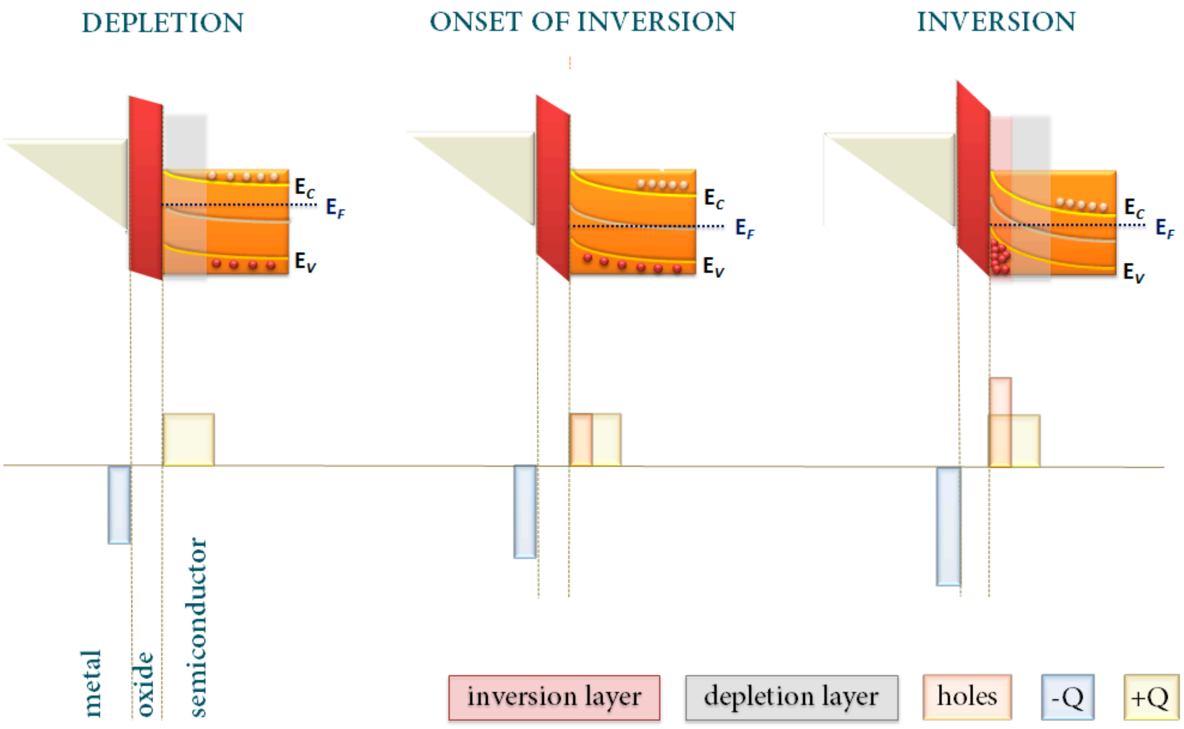

Scheme S1. The energy band and the charge density diagram of the Al/ReO_3@ReS_2/ITO memcapacitor under different bias conditions.

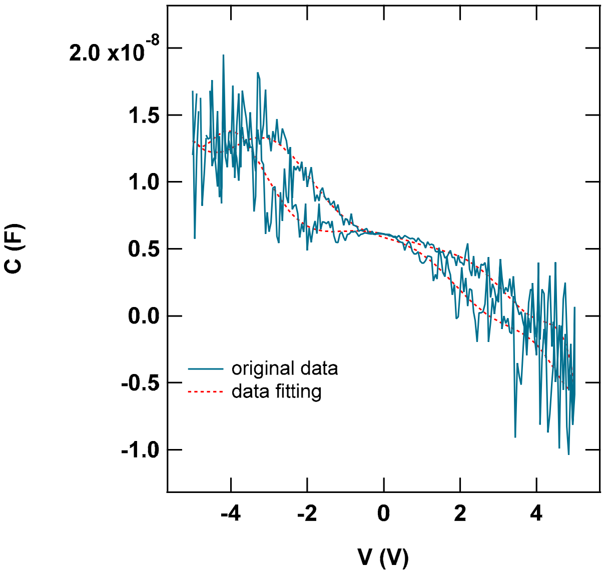

Figure S13. The C vs V curve under scan frequency of 1 kHz fitted with polynomial function of $y= a_0 + a_1x + a_2x^2 + \ldots + a_{n-1}x^{n-1}$.

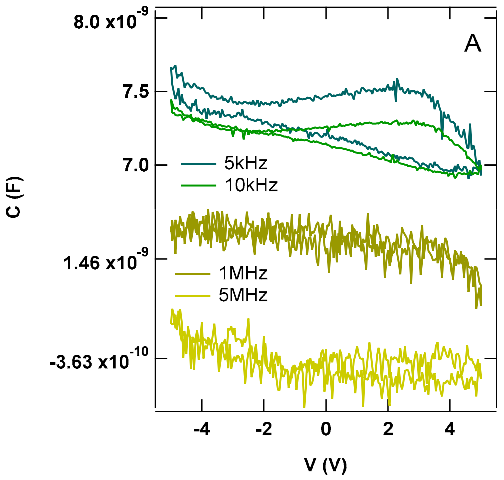

(**a**)

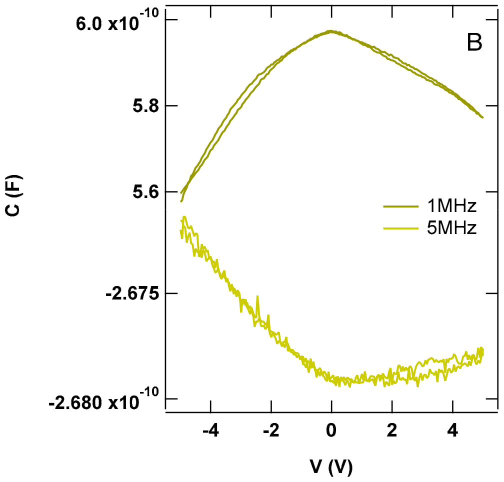

(**b**)

Figure S14. The C vs V curves of the ITO/ReO_3@ReS_2/Al memcapacitor under frequency range: (**a**) from 5 kHz to 5 MHz, and (**b**) at 1 and 5 MHz. Sweeping voltage range: ±5 V, and I_{cc}: 1×10-2 A.

2.6. Equation S6

$$C_{LF} = C_{HF} = \frac{1}{\frac{1}{C_{OX}}+\frac{x_d}{\varepsilon}} \quad for \quad V_{FB} \leq V \leq V_T \tag{6}$$

where,

$C_{LF,HF}$ low and high frequency capacitance

C_{OX} oxide layer capacitance

xd depletion layer width

ε permittivity

V_{FB} flat band voltage

V_T threshold voltage

2.7. Equation S7

$$C_{LF} = C_{ox} \text{ and } C_{HF} = \frac{1}{\frac{1}{C_{OX}}+\frac{x_{d,max}}{\varepsilon}} \quad for \quad V \geq V_T \tag{7}$$

where,

$C_{LF,HF}$ low and high frequency capacitance

C_{OX} oxide layer capacitance

xd,max depletion layer width at maximum

ε permittivity

V_T threshold voltage

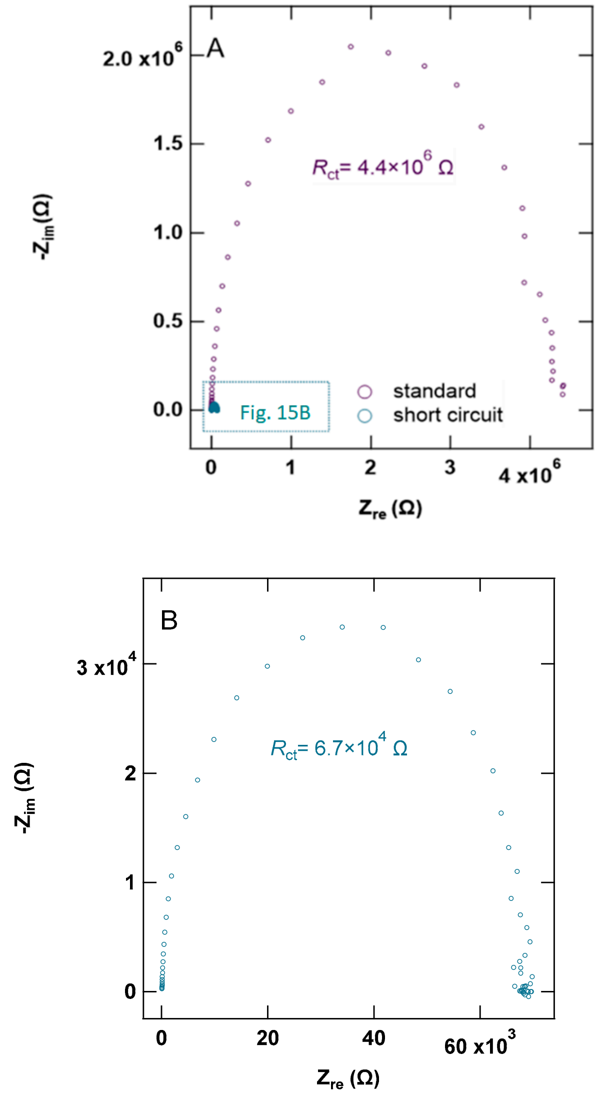

Figure S15. Nyquist plot of the impedance spectra of the (**A**) ITO/ReO_3@ReS_2/Al and (**B**) inset of (**A**) (short circuited device), acquired in the frequency range from 1×10^{-2} to 1×10^{5} Hz.
